# Multimodality imaging using SPECT/CT and MRI and ligand functionalized ^99m^Tc-labeled magnetic microbubbles

**DOI:** 10.1186/2191-219X-3-12

**Published:** 2013-02-25

**Authors:** Åsa A Barrefelt, Torkel B Brismar, Gabriella Egri, Peter Aspelin, Annie Olsson, Letizia Oddo, Silvia Margheritelli, Kenneth Caidahl, Gaio Paradossi, Lars Dähne, Rimma Axelsson, Moustapha Hassan

**Affiliations:** 1 Department of Clinical Science, Intervention and Technology (CLINTEC), Division of Medical Imaging and Technology, Karolinska Institutet, 141 86, Stockholm, Sweden; 2 Experimental Cancer Medicine (ECM), Department of Laboratory Medicine, Karolinska Institutet, 141 86, Stockholm, Sweden; 3 Department of Radiology, Karolinska University Hospital Huddinge, 141 86, Stockholm, Sweden; 4 Surflay Nanotec GmbH, 12489, Berlin, Germany; 5 Department of Medical Physics, Karolinska University Hospital Huddinge, 141 86, Stockholm, Sweden; 6 Department of Chemical Sciences and Technologies, University of Rome Tor Vergata, 00133, Rome, Italy; 7 Department of Molecular Medicine and Surgery, Karolinska Institutet, 171 76, Stockholm, Sweden; 8 Department of Clinical Physiology, Karolinska University Hospital Solna, 171 76, Stockholm, Sweden; 9 Department of Nuclear Medicine, Karolinska University Hospital Huddinge, 141 86, Stockholm, Sweden; 10 Clinical Research Center (KFC, Novum), Karolinska University Hospital Huddinge, 141 86, Stockholm, Sweden

**Keywords:** SPECT/CT, MRI, ^99m^Tc, Microbubbles, SPION, Multimodality imaging, Biodistribution

## Abstract

**Background:**

In the present study, we used multimodal imaging to investigate biodistribution in rats after intravenous administration of a new ^99m^Tc-labeled delivery system consisting of polymer-shelled microbubbles (MBs) functionalized with diethylenetriaminepentaacetic acid (DTPA), thiolated poly(methacrylic acid) (PMAA), chitosan, 1,4,7-triacyclononane-1,4,7-triacetic acid (NOTA), NOTA-super paramagnetic iron oxide nanoparticles (SPION), or DTPA-SPION.

**Methods:**

Examinations utilizing planar dynamic scintigraphy and hybrid imaging were performed using a commercially available single-photon emission computed tomography (SPECT)/computed tomography (CT) system. For SPION containing MBs, the biodistribution pattern of ^99m^Tc-labeled NOTA-SPION and DTPA-SPION MBs was investigated and co-registered using fusion SPECT/CT and magnetic resonance imaging (MRI). Moreover, to evaluate the biodistribution, organs were removed and radioactivity was measured and calculated as percentage of injected dose.

**Results:**

SPECT/CT and MRI showed that the distribution of ^99m^Tc-labeled ligand-functionalized MBs varied with the type of ligand as well as with the presence of SPION. The highest uptake was observed in the lungs 1 h post injection of ^99m^Tc-labeled DTPA and chitosan MBs, while a similar distribution to the lungs and the liver was seen after the administration of PMAA MBs. The highest counts of ^99m^Tc-labeled NOTA-SPION and DTPA-SPION MBs were observed in the lungs, liver, and kidneys 1 h post injection. The highest counts were observed in the liver, spleen, and kidneys as confirmed by MRI 24 h post injection. Furthermore, the results obtained from organ measurements were in good agreement with those obtained from SPECT/CT.

**Conclusions:**

In conclusion, microbubbles functionalized by different ligands can be labeled with radiotracers and utilized for SPECT/CT imaging, while the incorporation of SPION in MB shells enables imaging using MR. Our investigation revealed that biodistribution may be modified using different ligands. Furthermore, using a single contrast agent with fusion SPECT/CT/MR multimodal imaging enables visualization of functional and anatomical information in one image, thus improving the diagnostic benefit for patients.

## Background

Multimodality imaging is becoming an essential tool in diagnostics. Multimodality imaging provides functional and anatomical data from different imaging techniques such as single-photon emission computed tomography (SPECT)/magnetic resonance imaging (MRI), SPECT/computed tomography (CT) or positron emission tomography (PET)/MRI to obtain the highest readout from *in vivo* examination and hence increase clinical efficacy.

Poly(vinyl alcohol) (PVA) microbubbles (MBs) functionalized with ligands are currently undergoing several investigations worldwide as a potential contrast agent [1-5] to facilitate diagnosis and visualize physiological changes in diseases such as cancer, stroke, metabolic disorders, inflammation, and ischemia [2,6-8]. The functionalization of MBs by different ligands to incorporate superparamagnetic iron oxide nanoparticles (SPION) makes them visible by multimodality imaging techniques, thus enabling their use for diagnostic purposes [9]. Incorporating SPION in the shells of MBs facilitates their visualization by MRI, while functionalization of MBs with ligands (which can incorporate radioactive tracers) facilitates visualization using SPECT/CT. Imaging techniques using SPECT/CT and MRI as less invasive approaches for testing new drugs offer a number of benefits. These include reducing the number of laboratory animals used for research, increasing reproducibility, and saving time and money [10]. Moreover, imaging techniques can visualize the distribution and elimination of the contrast agents *in vivo*, which can be utilized as a diagnostic tool to differentiate between healthy and pathological tissues. The ligand-functionalized MBs might therefore be of interest for diagnostic imaging, including the diagnosis of cancer. MBs also have the capability to act as a delivery vehicle for drugs or cytostatic agents [11-13]. Several attempts have been made to improve drug delivery systems in order to enhance stability, pharmacokinetics, bioavailability, and biodistribution for several compounds [14]. Moreover, drug delivery systems may minimize the adverse effects of drugs by directing them to the target organs.

SPECT has become a powerful tool for imaging the biodistribution of molecules labeled with radioactive isotopes such as ^99m^Tc or ^123^I as a step in the early stages of drug development [15-20]. SPECT offers functional information, while CT and MRI offer anatomical and physiological details. Combined SPECT/CT has improved diagnostics since it enhances both sensitivity and specificity. SPECT/CT has shown superiority in several medical fields including the diagnosis of carcinoids, brain tumors, lymphomas, prostate cancers, bone lesions, and infections [21]. SPECT/CT has also led to a better evaluation of therapeutic outcome in cardiovascular as well as hepatic and renal impairment patients [17,21].

The chelating ligand diethylenetriaminepentaacetic acid (DTPA) in its pure form is rapidly cleared through urine in healthy kidneys and is therefore often used to quantify renal impairment [22]. ^99m^Tc-DTPA kits are commercially available for renal imaging and are used to measure renal function and glomerular filtration rate. In humans, the biological half-life of DTPA is 1 to 2 h and renal uptake is 7% [14]. However, scant information is available about the biodistribution and elimination of DTPA-functionalized MBs.

In order to study the effect of ligands on biodistribution, several ligands have been chosen to functionalize the MBs. DTPA, 1,4,7-triacyclononane-1,4,7-triacetic acid (NOTA), chitosan, thiolated poly(methacrylic acid) (PMAA) and plain MBs were labeled with ^99m^Tc and studied. These ligands have the following properties: chitosan is a linear polysaccharide with a backbone characterized by a random distribution of β-(1-4)-linked d-glucosamine and *N*-acetyl-d-glucosamine units. Chitosan is biocompatible, is known to have good chelating capacity for metal ions, and has previously been used as a nonviral gene delivery system to the lungs [23-25]. NOTA is a monoreactive ligand commonly used for tracer attachment in PET imaging and has been used for imaging of tumors in the pancreas, stomach, and adrenals [26]. PMAA may be used as a ligand on MBs to further improve the MBs coupling to other moieties. It has been used to control shell functionality of microspheres due to its biocompatibility [27,28].

SPION was incorporated between the layers of PVA MBs followed by ligand coupling with DTPA or NOTA to enable the multimodality imaging of MBs using SPECT and MRI. In the current study, we imaged NOTA-SPION and DTPA-SPION MBs using SPECT/CT and MRI to visualize their biodistribution since ^99m^Tc is also incorporated into the PVA matrix of MBs.

Little is known about the function, distribution, and elimination of the ligands used to functionalize MBs in this study, despite their previous use in the medical field. The aim of the present study is to investigate the distribution and elimination of ligand-functionalized MBs using SPECT/CT and MRI in order to further develop MBs as contrast agent for cancer diagnostics and detection of inflammation, and/or to evaluate their use in multimodality imaging which might improve the diagnostic methods.

## Methods

MBs were functionalized with several ligands, and their *in vivo* distribution in rats was investigated using SPECT/CT and MRI. The following aminoguanidine (AG) MBs were studied: AGMBs (plain MBs), AGMBs/DTPA (DTPA MBs), AGMBs/PMAA (PMAA MBs), AGMBs/NOTA (NOTA MBs), AGMBs/NOTA SPION (NOTA-SPION MBs), and AGMB/DTPA-SPION (DTPA-SPION MBs) obtained from Surflay Nanotec GmbH, Berlin, Germany. Moreover, chitosan MBs were studied and obtained from the Department of Chemical Sciences and Technologies, University Tor Vergata, Rome, Italy.

### The formation of microbubbles

PVA-shelled MB synthesis has been described elsewhere [29]. The synthesis can be summarized as follows: 2 g PVA at 70 K (P1763, Sigma-Aldrich Chemie GmbH, Munich, Germany) was added to 100 mL of Milli-Q water (Millipore Co., Billerica, MA, USA). The suspension was stirred at 80°C to allow PVA dissolution. Approximately 0.19 g of sodium metaperiodate was added to the solution, and the mixture was stirred for 1 h at 80°C. The solution was cooled to room temperature, with continuous stirring to avoid the formation of a PVA film. The cross-linking reaction was then carried out under vigorous stirring at room temperature for 2 h at 8,000 rpm by an Ultra-Turrax T-25 (IKA Works, Inc., Wilmington, NC, USA) equipped with a Teflon-coated tip. Floating MBs were separated from solid debris and extensively washed with Milli-Q water.

### Aminoguanidine coupling

The MB suspension was centrifuged at 30×*g* for 1 h followed by the removal of the subnatant. AG (5mg/mL) in 0.1 M Hepes buffer (pH 8) was added, and the suspension was kept on the shaker for 1 day.

**Figure 1:**
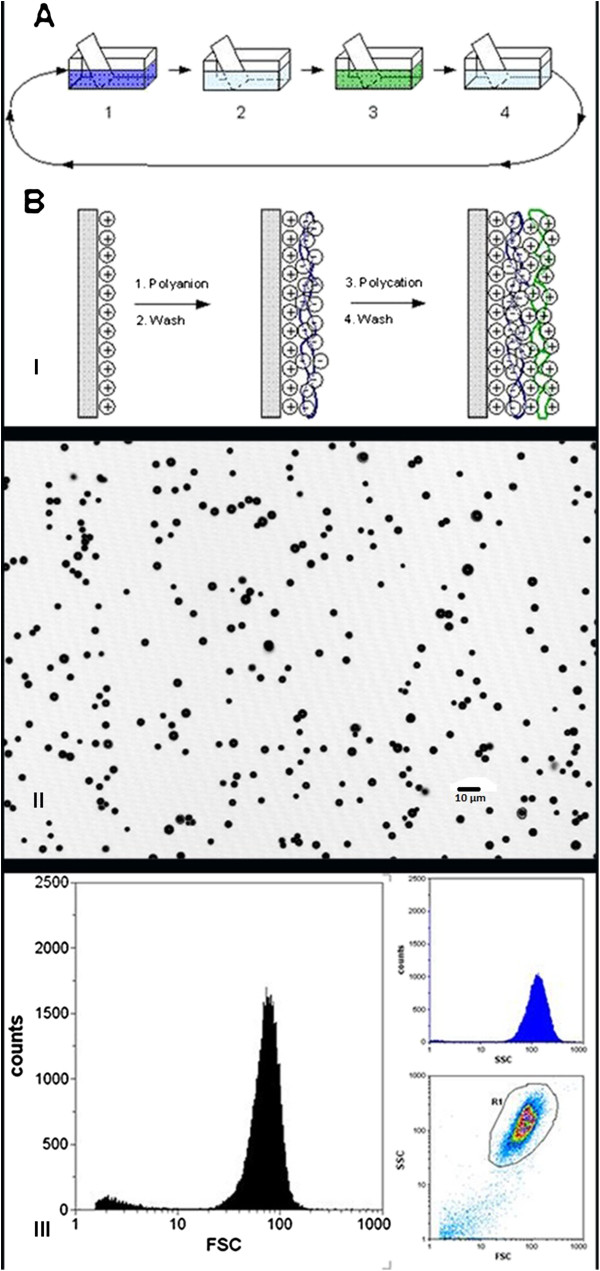
**Production of MBs and quality control.** The MBs were washed in several steps (part I, **A**). MBs in the upper phase were separated by centrifugation, isolating the plain MBs with a positively charged surface. These MBs were then functionalized by different ligands using the layer-by-layer technique (part I, **B**). As quality control, DTPA-SPION MBs were visualized by the confocal laser microscope (Leica, resolution 550 μm × 550 μm; part II), and flow cytometry (Partec CyFlow ML flow cytometer) was used to determine the size distribution of DTPA-MBs (part III) using forward scattering versus side scattering dot plot.

Afterward, the MBs were washed three times. After each wash, the MBs in the upper phase were separated by centrifugation (30×*g*, 1 h), and the subnatant was removed. Plain MBs with a positively charged surface (amino groups) were obtained. The charged surface is necessary to functionalize MBs by different ligands including DTPA, PMAA, NOTA, NOTA-SPION, and DTPA-SPION (part I of Figure 1). MBs were visualized using confocal laser microscopy at a resolution of 550 μm × 550 μm (part II of Figure 1) (Leica TCS SPE, Leica Microsystems, Wetzlar, Germany). Size distribution was determined using flow cytometry (Partec CyFlow ML, Partec GmbH, Münster, Germany). Analysis was carried out using forward scattering versus side scattering. Part III of Figure 1 shows DTPA-SPION MBs.

### Preparation of DTPA MBs

**Figure 2:**
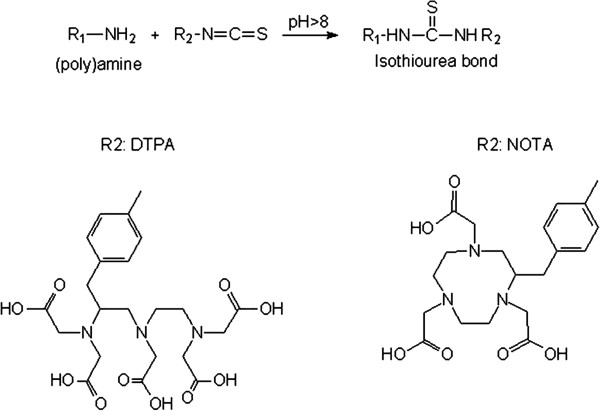
**Chemical structures of the ligands DTPA and NOTA.** The polyamines with the chelating ligand DTPA or NOTA react with isothiocyanates at pH 8 to form a stable isothiourea bond in the molecule containing NOTA or DTPA.

The DTPA-isothiocyanate (0.2 mg DTPA/10^8^ MBs) was added to a suspension of AGMBs in 10 mM NaHCO_3_ at pH 8. The reaction mixture was shaken overnight, washed, and separated by centrifugation (30×*g*, 40 min). Polyamines in the top layer of the MB surface react with isothiocyanates at pH 8 to form a stable isothiourea bond in molecules containing DTPA to be functionalized onto MBs (Figure 2).

### Preparation of NOTA MBs

NOTA-isothiocyanate (0.2 mg NOTA/10^8^ MBs; Figure 2) was added to a suspension of AGMBs in 10 mM NaHCO_3_ at pH 8. The reaction mixture was shaken overnight and washed as described previously.

### Chitosan oxidation

The metaperiodate ion, IO_4_^−^, cleaves to the C2-C3 bond of the glucosamine residue of chitosan, leading to the formation of a dialdehyde with the elimination of an ammonia molecule. However, the *N*-acetylated amino groups (in small amounts) at the C2 position of the chitosan backbone (15%) prevent the oxidation cleavage by metaperiodate. For the oxidation reaction, chitosan was dissolved in 1% (*w*/*v*) double-distilled water/HCl at pH 4.5 and was thereafter mixed with a given amount of NaIO_4_ (dark condition). A twofold molar excess of chitosan in relation to NaIO_4_ was used. After 24 h, the solution was dialyzed (dialysis membrane, MWCO of 2,000) against deionized water (pH 4.5), lyophilized, and stored as a freeze-dried powder. The oxidation that leads to chain depolymerization as a side reaction increased the low solubility of chitosan in water and prevented cluster formation of the MBs. The freeze-dried periodate-oxidized chitosan was dissolved in D_2_O (5% *w*/*w* trimethylsilanol (TMS)) at a concentration of 5 mg/mL. The resulting solution was subjected to ^1^H-NMR examination, and the degree of oxidation was calculated considering the integral of the C2 resonance at 3.00 ppm, taking as reference the signal of the trimethyl group of TMS at 0.0 ppm (9H, TMS trimethyl group).

### Oxidized chitosan coupling to the surface of MBs

**Figure 3:**
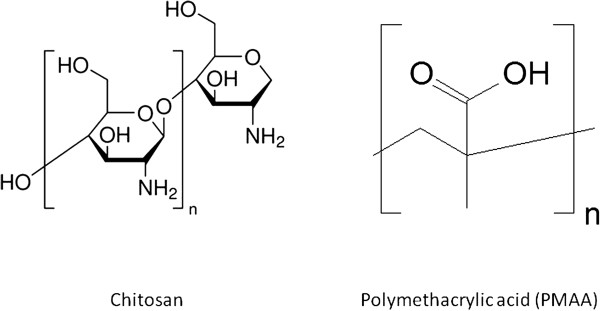
Chemical structures of the ligands chitosan and PMMA.

The oxidized chitosan (Figure 3) is conjugated to MBs by an acetylization procedure. The polymer is dissolved in Milli-Q water to a concentration of 1% (*w*/*v*). A sample of 20-mg aqueous MB suspension was added, and the final volume was adjusted to 10 mL. The pH was carefully adjusted to 3.0 with 0.1 M HCl. The resulting suspension was stirred in the dark for 5 days at room temperature followed by extensive washing in separatory funnels and stored in Milli-Q water at 4°C*.*

### Preparation of PMAA MBs

The AGMBs were coated in standard PMAA (MW approximately 1 × 10^5^ g/mol; Figure 3) solution at pH 6.2. The reaction took approximately 1 day, and the reaction mixture was washed as described previously under DTPA preparation.

### Preparation of magnetic microbubbles with NOTA (NOTA-SPION MBs) or DTPA (DTPA-SPION MBs)

The AGMBs were coated by layers of polystyrene sulfonate (PSS), polyethyleneimine (PEI), and polyallylamine (PAH) as PSS 70,000/PEI 25,000/PSS/PEI/PSS/PAH. To this, base magnetite/PAH/magnetite/PAH/magnetite/PAH was attached using the layer-by-layer technique [30]. When the first magnetic layer had been attached to the bubbles, they were washed and separated in a magnetic field. This procedure was repeated for each layer. The NOTA or the DTPA was coupled covalently to the outmost PAH layer as described previously to produce NOTA-SPION MBs or DTPA-SPION MBs. The incorporation of SPION was determined to be 3.8 mg Fe/10^9^ MBs/mL.

### ^99m^Tc-MB synthesis

The reduced ^99m^Tc is chemically reactive and can be reacted with a chelating agent to become a ^99m^Tc-chelate. The chemical group –COO^-^ is the electron donor of DTPA and NOTA and NH_2_ is the electron donor of chitosan as follows:

$$2^{\text{99m}}\text{TcO}{-}_{4}+{\text{16H}}^{+}\;{\text{3Sn}}^{\text{2+}}\Leftrightarrow 2^{99\text{m}}{\text{Tc}}^{\text{4+}}+{\text{3Sn}}^{\text{4+}}+{\text{8H}}_{2}\text{O}\;$$

Sodium pertechnetate (^99m^Tc-NaTcO_4_) was obtained from a ^99^Mo/^99m^Tc generator. ^99m^Tc was obtained using the reducing agent stannous chloride SnCl_2_·2H_2_O (Sigma-Aldrich Chemie GmbH, Munich, Germany). MBs (0.2 mL of 10^8^ MBs/mL) were diluted with 0.3 mL of isotonic saline in 2-mL Eppendorf tubes (Eppendorf AG, Hamburg, Germany). SnCl_2_·2H_2_O (0.5 mL) was added to the MB solution at room temperature. ^99m^Tc-NaTcO^4^ (0.5 mL) with an activity of 1,000 to 1,500 MBq (Dose Calibrator CRC®-15 PET, Capintec Inc., Pittsburgh, PA, USA) was added to the ligand-functionalized MB-SnCl_2_ solution for reduction reaction (5 min) at pH 5. The Eppendorf tubes were centrifuged (500 rpm, 4 min, Hettich Universal 16, Andreas Hettich GmbH & Co. KG, Tuttlingen, Germany), and the subnatant was removed. After centrifugation, isotonic saline was added, and the procedure was repeated until radioactivity in the subnatant was equal to or less than 5% of the activity of MBs in the upper phase. To evaluate the stability of labeled MBs, 0.2 mL (10^9^ MBs/mL) of DTPA-functionalized MBs and plain MBs were labeled as previously described using 60 MBq. The MBs were washed until less than 5% remained in the subnatant. The MBs were resuspended in saline, and the radioactivity of the subnatant was followed for four ^99m^Tc half-lives to determine the leakage of ^99m^Tc from the MBs.

### Analysis by instant thin-layer chromatography

Instant thin-layer chromatography (ITLC) was carried out using an ITLC-SG silica gel (ITCL^TM^ SG, Life Sciences Advanced Technology Inc., St. Petersburg, FL, USA) impregnated strip of 5 cm × 20 cm and acetone as a mobile phase for 30 min. Equal volumes at 20 μL of ^99m^Tc-labeled MBs and ^99m^Tc-pertechnetate were placed on the ITLC-SG strip and developed using acetone. ITLC was evaluated by a gamma camera detector (Siemens Symbia Vega, Siemens AG, Erlangen, Germany). Cobalt pens were used as markers at *R*_*f*_ 0 (MBs), *R*_*f*_ 50 (0), and *R*_*f*_ 100 (^99m^Tc-NaTcO_4_). The strip was recorded for 1 min with cobalt pens and 1 min without cobalt pens. The ITLC strip was thereafter cut into eight pieces which were numbered, and activity was measured in each piece by the CRC®-15 PET dose calibrator (Capintec, Inc.).

### Animal studies

The study was approved by the Stockholm Southern Ethical Committee on Animal Research and was performed in accordance with Swedish Animal Welfare law. Male Sprague Dawley rats weighing 300 ± 50 g were purchased from Charles River (Charles River Laboratories, Sulzfeld, Germany). After arrival, the animals were allowed to acclimatize for at least 1 week in the animal facility before the start of the experiment. The animals were given access to food and water *ad libitum*, 12-h light/dark cycle, controlled humidity (55 ± 5%), and temperature (21 ± 2°C). The rats were anesthetized using an intraperitoneal injection of pentobarbital (sodium pentobarbital 60 mg/mL, APL Kungens Kurva, Sweden) at a dose of 50 mg/kg. Hybrid imaging was performed following an intravenous injection of 0.5 mL MBs via the tail vein on freely breathing rats using SPECT/CT, followed by 3-T MRI. Animals were injected with 0.5 mL ^99m^Tc-labeled plain, DTPA, chitosan, PMAA, NOTA, NOTA-SPION, or DTPA-SPION MBs. The rats that were injected with plain MBs and MBs functionalized with PMAA, chitosan, or DTPA received a dose of 10 to 20 MBq, while rats to be imaged with MRI and SPECT and followed for 24 h received a dose of 100 to 150 MBq. Two rats were used for a pilot study to set up the imaging parameters, and two rats were used to study each ligand-functionalized MB sample. Free sodium pertechnetate (^99m^Tc-NaTcO_4_) was obtained from the ^99^Mo/^99m^Tc generator, injected, and imaged as a reference. ^99m^Tc-DTPA without attachment to MBs was also injected as a reference to visualize the DTPA distribution compared to MBs functionalized by DTPA.

In the second part of the study, rats were injected with plain MBs and MBs functionalized with PMAA, NOTA, chitosan, or DTPA. Each animal received a dose of 10 to 20 MBq (*n* = 4 for each MB): two were sacrificed at 11.7 min (time for regions of interest (ROI) measurement), and two were sacrificed 1 h post injection. Rats were injected with NOTA-SPION MBs (*n* = 6) or DTPA-SPION MBs (*n* = 6) at a dose of 100 to 150 MBq. Two rats per MB were sacrificed at 11.7 min, two were sacrificed at 1 h, and two were sacrificed at 24 h. The liver, lungs, spleen, bladder, kidneys, and blood were removed, and radioactivity was measured.

### Nuclear medicine imaging

SPECT/CT imaging was performed using a Siemens Symbia True Point 16 hybrid system (Symbia® Siemens, Erlangen, Germany) with parallel hole LEHR collimators.

### Dynamic planar scintigraphy and SPECT/CT

Initially, the animals were placed in prone position in the gamma camera. The ^99m^Tc-labeled functionalized MBs, ^99m^Tc-pertechnetate, and ^99m^Tc-TechneScan® DTPA (^99m^Tc-DTPA kit) (Mallinckrodt, Hazelwood, MO, USA) were injected intravenously into the tail vein during dynamic planar scintigraphy (two frames per second) for a total duration of 11.4 to 11.7 min. The matrix size used was 256 × 256 with a suitable energy window for ^99m^Tc (15% wide). To follow the distribution and clearance of SPION MBs using MRI and SPECT/CT, the animals injected with NOTA-SPION MBs and DTPA-SPION MBs were additionally imaged using dynamic planar scintigraphy 24 h post injection. Directly after dynamic scintigraphy, SPECT was performed for all animals injected with ^99m^Tc-labeled ligand-functionalized MBs, ^99m^Tc-DTPA kit, and ^99m^Tc-NaTcO_4._ Animals injected with NOTA MBs, NOTA-SPION MBs, and DTPA-SPION MBs were also imaged 24 h post injection using SPECT/CT. The SPECT parameters used were 128 × 128 matrix, 64 projections over 360°, noncircular orbit, step-and-shoot mode. The duration of each projection was 60 s. The acquisition time required for SPECT was 32 min. Immediately after the SPECT acquisition, a CT scan was performed with a tube current of 110 kV, quality reference milliampere second was 160 mA s, and modulated with CareDose4D®; rotation time was 0.6 s, and pitch was 1.0. Subsequently, the CT data were reconstructed with a slice thickness of 0.75 mm and a “B70s sharp” kernel. Reconstruction of the SPECT data and evaluation of the resulting images were made on a Hermes workstation (Hermes Medical Solutions AB, Stockholm, Sweden). Iterative reconstruction was made with ordered subset expectation maximization (four iterations, eight subsets, including resolution recovery). A 3D Gaussian post filter (0.8 cm FWHM) was finally applied.

### Evaluation

ROIs were outlined for the organs on the dynamic images, and counts were measured in each ROI. No correction for radioactive decay was made due to the short time interval. The number of counts in each organ was compared to the total counts in the whole animal to obtain the percentage of distribution to each organ. Interpretation of fused SPECT/CT images was made in three orthogonal planes. To establish the values obtained from ROIs, animals were sacrificed, organs were removed, and radioactivity was measured. The radioactivity in organs was compared to the total injected dose.

### Magnetic resonance imaging

Rats injected with NOTA-SPION MBs and DTPA-SPION MBs were first imaged using SPECT/CT and then brought to the 3-T MRI scanner (Siemens Trio, Siemens, Erlangen, Germany) for imaging. The animals were put head first in prone position in an extremity coil. Imaging was performed using a gradient echo T2* sequence to obtain images with a fixed repetition time of 2,000 ms and 12 stepwise increasing echo times (TEs) of 2 to 22.9 ms. Field of view was 250 mm with a phase encoding of 59.4% and a slice thickness of 3 mm. Circular ROIs were placed in the respective organs, and the negative logarithmic values of signal intensities at different TEs were plotted versus respective TE values followed by calculation of T2*. In the organs with short T2* (i.e., the liver after injection of NOTA-SPION MBs and DTPA-SPION MBs), the calculations were based on fewer measurement points, excluding those with long TEs where full transaxial relaxation had already occurred. The rats were imaged using 3-T MRI before injection of MBs (reference value), approximately 2 and 24 h post injection of the ^99m^Tc-labeled NOTA-SPION MBs and DTPA-SPION MBs.

## Results and discussion

### Results

**Figure 4:**
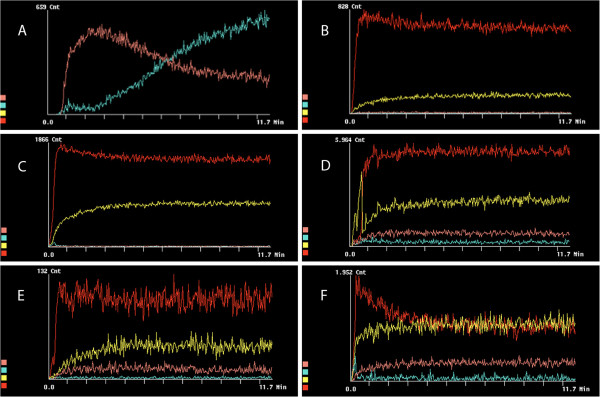
**Dynamic distribution in rat organs within the first 11.7 min post injection of**^**99m**^**Tc-DTPA kit and**^**99m**^**Tc-labeled MBs.** Designated colors for organs: lungs (red), liver (yellow), bladder (turquoise), kidneys (maroon). (**A**) DTPA kit. (**B**) Plain MBs. (**C**) DTPA MBs. (**D**) Chitosan MBs. (**E**) PMAA MBs. (**F**) NOTA MBs.

The ^99m^Tc labeling yield of MBs was dependent on the functional ligand attached to the MBs. The labeling yields (expressed as percentage of the amount of radioactivity at the start) after ^99m^Tc labeling, purification, and washing steps were in the following order: MBs functionalized with DTPA, 60%; MBs functionalized with PMAA, 45%; MBs functionalized with NOTA, 42%; and MBs functionalized with chitosan, 25%; while the labeling of plain MBs yielded about 20%. The labeling yield was 85% for MBs with DTPA-SPION and 52% for MBs with NOTA-SPION. No precipitate was observed after ^99m^Tc labeling and washing. The radiochemical purity of the washed and purified MBs was equal to or above 95% as determined by the dose calibrator. These results were confirmed using ITLC assay. Moreover, *in vitro* studies showed that labeled MBs resuspended in saline were stable (no significant leakage was detected). Considering the radioactivity decay, 6.25% of the initial radioactivity should remain. After 24 h (four half-lives), we detected 5.4% of the initial radioactivity in MBs, indicating the stability of the labeling. Figure 4 represents the dynamic distribution of ^99m^Tc-labeled MBs with different ligands; the values are summarized in Table 1. As can be seen in Figure 4A, a rapid uptake by the kidneys occurs during the first 2 min followed by a slow decline up to 11.7 min. After 2 min, the radioactivity was eliminated from the kidneys and accumulated in the bladder. Figure 4B shows the dynamic distribution of plain MBs: the uptake was about 69% in the lungs, 25% in the liver, and less than 5% in the kidneys (Table 1). DTPA MBs (Figure 4C) were mainly distributed to the lungs (62%), while 28% of the uptake was observed in the liver, and less than 10% was found in the kidneys (Table 1). Uptake of chitosan MBs (Figure 4D) was observed mainly in the lungs (64%), while about 28% was distributed to the liver, and <10% was observed in both bladder and kidneys (Table 1). Figure 4E illustrates the dynamic distribution of MBs functionalized with PMAA: 58% of the injected radioactive tracer was distributed to the lungs within the first minute, while the distribution to the liver reached 34% within the first 2 min, and less than 10% was observed in the kidneys (Table 1). NOTA MBs (Figure 4F) were rapidly distributed to the lungs, with the highest uptake within less than 1 min (43%). Within 3 min, the activity had declined in the lungs, and it reached a steady state at 3 min post injection. Distribution to the liver (37%) was reached within the first minute; about 8% was found in the kidneys, and less than 5% was found in the bladder (Table 1). These results were confirmed by removing the organs and measuring the radioactivity in each organ at 11.7 min and 1 h for all MBs, and at 24 h post injection of NOTA MBs and DTPA-SPION MBs (Table 1). The results are expressed as (ratio of radioactivity in organ/injected dose × 100). Moreover, less than 3% of the total injected dose was detected in the blood, and no radioactivity was detected in the bladder/urine.

**Table 1:** Percentage of radioactivity found in organs expressed as ROI/total counts and radioactivity/injected dose

**MBs**	**ROI measurements as percentage of the total counts obtained from dynamic imaging**			**Total radioactivity in organs as percentage of injected dose**					
				**11.7 min**			**1 h**		
	Lungs	Liver	Kidneys	Lungs	Liver	Kidneys	Lungs	Liver	Kidneys
Plain	69	25	3	67	32	2	59	36	8
DTPA	62	28	7	66	25	5	52	43	6
Chitosan	64	28	3	58	22	2	51	39	5
PMAA	58	34	8	57	35	4	48	43	7
NOTA	43	37	8	50	40	3	47	45	6
NOTA-SPION	56	24	8	51	23	4	60	20	6
NOTA-SPION(24 h, PI)	8	50	18				6^a^	54^a^	16^a^
DTPA-SPION	93	4	1	89	6	1	65	38	7
DTPA-SPION(24 h, PI)	25	40	40				21^a^	37^a^	41^a^

The distribution of radioactivity to different organs was expressed either as ROI measurements (as percentage of the total counts obtained from dynamic imaging at 11.7 min) or as radioactivity measured in organs (as percentage of the total injected dose after 11.7 min and after 1 h post injection). ^a^Values measured at 24 h.

**Figure 5:**
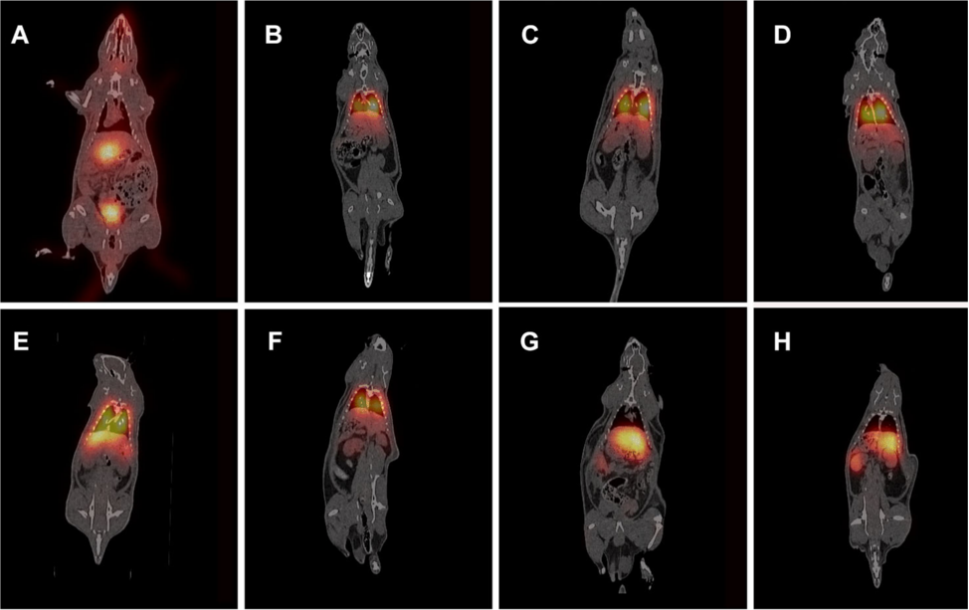
**Coronal SPECT/CT fusion images illustrating the uptake of**^**99m**^**Tc-pertechnetate and**^**99m**^**Tc-labeled MBs in rats.** (**A**) ^99m^Tc-pertechnetate, (**B**) plain MBs, (**C**) DTPA MBs, (**D**) chitosan MBs, (**E**) PMAA MBs, and (**F**) NOTA MBs, 1 h post injection. (**G**) NOTA MBs, liver view, 24 h post injection. (**H**) NOTA MBs, kidney view, 24 h post injection.

Figure 5 illustrates the fusion SPECT/CT images for free ^99m^Tc-pertechnetate and MBs functionalized with different ligands. The distribution of free ^99m^Tc-pertechnetate (Figure 5A) post injection shows that the radioactivity was distributed from highest to lowest in the following order: stomach, bladder, thyroid gland, thymus, liver, and mouth mucosal membrane. The distribution of plain MBs was mainly to the lungs and, to a lesser extent, to the liver, spleen, and kidneys (Figure 5B).

**Figure 6:**
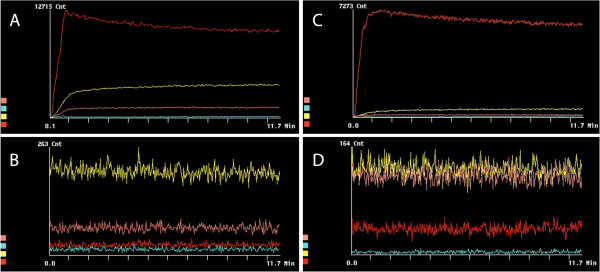
**Dynamic distribution in rat organs within the first 11.7 min and 24 h post injection of**^**99m**^**Tc-labeled NOTA-SPION and DTPA-SPION MBs.** Designated colors for organs: lungs (red), liver (yellow), bladder (turquoise), and kidneys (maroon). (**A**) NOTA-SPION MBs during the first 11.7 min post injection. (**B**) NOTA-SPION MBs 24 h post injection. (**C**) DTPA-SPION MBs during the first 11.7 min post injection. (**D**) DTPA-SPION MBs 24 h post injection.

The addition of DTPA as a ligand to MBs affects the distribution, as can be seen in Figure 5C. DTPA MBs were mostly observed in the lungs, followed by the liver and spleen. No distribution to other organs could be detected. Similar distribution was seen after the administration of chitosan MBs (Figure 5D). PMAA MBs were also distributed mainly to the lungs and liver (Figure 5E). However, a higher distribution was seen in the liver compared with that observed for DTPA MBs and chitosan MBs. NOTA MBs were distributed mainly to the lungs and, to a lesser extent, to the liver, followed by the spleen, kidneys, and bladder (Figure 5F). After 24 h, NOTA MBs were mainly located in the liver, stomach, and kidneys (Figure 5G,H). NOTA MBs and NOTA-SPION MBs showed a different distribution pattern at dynamic imaging compared with the other MB types described in this study (Figures 4F and 6A). After 11.7 min, the highest uptake of NOTA-SPION MBs was observed in the lungs (56%), followed by the liver (24%), and, to a lesser extent, the kidneys (8%), and the bladder (<5%) (see Figure 6A). After 24 h post injection, the activity had been redistributed so that the main activity was located in the liver (50%) and, to a lesser extent, in the kidneys (18%), lungs (8%), and bladder (<5%) (see Figure 6B).

DTPA-SPION MBs showed a rapid and exclusive distribution to the lungs at 11.7 min post injection (93%) (Figure 6C). After 24 h, redistribution of radioactivity was observed in the liver (40%), kidneys (40%), and lungs (25%) as can be observed in Figure 6D.

**Figure 7:**
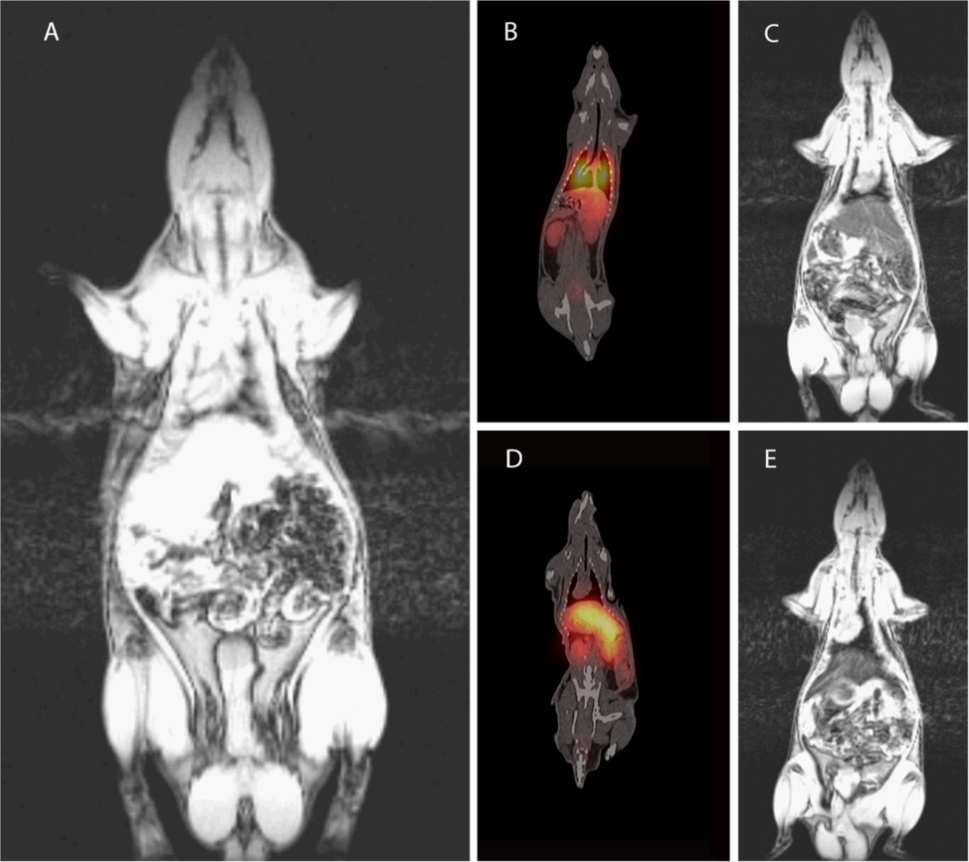
Coronal fusion SPECT/CT images and 3-T MRI T2* images illustrating the uptake of^**99m**^**Tc-labeled NOTA-SPION MBs in rats.** (**A**) 3-T MRI pre-injection. (**B**) SPECT/CT, 1 h post injection. (**C**) 3-T MRI, 2 h post injection. (**D**) SPECT/CT, 24 h post injection. (**E**) 3T MRI, 24 h post injection.

**Figure 8:**
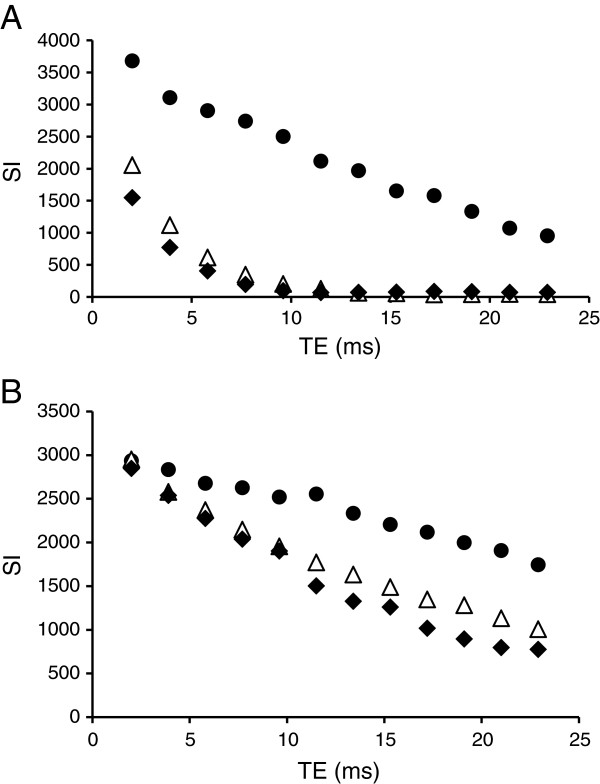
**MR signal decay in various organs.** MR signal decay in the liver (**A**) and in the kidneys (**B**) of the rat before (black circle), 2 h (triangle), and 24 h (black diamond) post injection of NOTA-SPION MBs and DTPA-SPION MBs based on ROI measurements.

**Figure 9:**
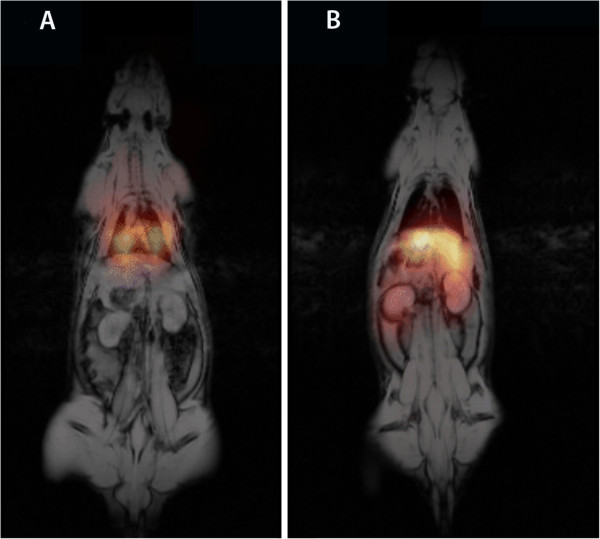
**Multimodality imaging using SPECT/MRI.** SPECT enables the functional visualization of MBs in the lungs 1 h post injection (**A**) and in the liver and kidneys 24 h post injection (**B**), while MRI enables the visualization of an anatomical image and SPION in the liver post injection and 24 h post injection (A and B).

Figure 7A shows MR image pre-injection. As shown in the fusion SPECT/CT image (Figure 7B), a high uptake was observed in the lungs, while a lesser uptake was seen in the liver, kidneys, and bladder. Because it is a dual contrast agent, the distribution of NOTA-SPION MBs to the liver could also be confirmed by MRI, in which the signal of the liver was markedly decreased 2 h post injection (Figure 7C) compared with that prior to injection (Figure 7A). After 24 h post injection, the radioactivity was redistributed mainly to the liver and spleen (Figure 7D) and, in a lower amount, to the kidneys. The accumulation in the liver was confirmed using MRI (Figures 7E and 8A). Based on the ROI measurements in both the liver and kidneys using MRI, the T2* of the liver decreased by 79% 2 h post injection and by 82% 24 h post injection (Figure 8A). A 50% T2* decrease was calculated 2 h post injection in the kidneys, while it had decreased by 64% 24 h post injection (Figure 8B). The SPECT and MR images were fused for rats injected with DTPA-SPION MBs (Figure 9), visualizing the concept of multimodality imaging using fusion SPECT-MRI where SPECT exclusively enables the visualization of MBs in the lungs 1 h post injection (Figure 9A) and in the liver and kidneys 24 h post injection (Figure 9B).

### Discussion

In the present study we successfully labeled the newly developed MBs with ^99m^Tc using DTPA, chitosan, PMAA, and NOTA. The labeling yield of MBs was affected by the attached ligand but was also seen in plain MBs. The highest labeling yield was observed in MBs functionalized with DTPA, followed by PMAA, and NOTA. The fact that plain MBs as well as PMAA and NOTA MBs could also be labeled by ^99m^Tc, but to a lesser extent, most probably indicates that ^99m^Tc was incorporated into the PVA matrix of the bubbles by nonspecific binding.

The distribution of MBs, as observed using dynamic planar scintigraphy and SPECT/CT, was affected by the attached ligands. DTPA, chitosan, and PMAA MBs were mainly distributed to the lungs and, to some extent, to the liver, while NOTA MBs were evenly distributed to the lungs and the liver. Moreover, uptake was observed in the kidneys after administration of PMAA MBs, NOTA MBs, and NOTA-SPION MBs. This is most probably due to free ^99m^Tc liberated *in vivo* from the MBs and eliminated mainly through the kidneys. In an *in vitro* test using labeled MBs resuspended in saline, a nonsignificant amount of ^99m^Tc had been released from the MBs 10 and 30 min and 1, 3, and 24 h after ^99m^Tc labeling. Another possible explanation for the activity observed in the kidneys is that some of the MBs might burst *in vivo* and fragments carrying ^99m^Tc might then be eliminated via the kidneys. After 24 h, the NOTA-SPION MBs were redistributed from the lungs to the liver and kidneys. Moreover, measuring the radioactivity in isolated organs after injecting different ^99m^Tc-labeled MBs was in good agreement with the results obtained from the gamma camera and SPECT.

SPECT/CT imaging of animals injected with NOTA-SPION MBs demonstrated major distribution to the lungs and, to a lesser extent, to the liver and kidneys, while the MBs had been redistributed mainly to the liver 24 h post injection. Actually, radioactivity was no longer visually observed in the lungs, while it was still visible in the kidneys and spleen. Co-registration of NOTA-SPION MBs using 3-T MRI assessed 2 and 24 h post injection confirmed a considerable increase in liver uptake with concomitant canceling of the MRI signal during the first 24 h. The uptake of ^99m^Tc- labeled DTPA-SPION MBs was observed in the lungs 1 h post injection using SPECT, while the redistribution to the liver and kidneys was seen on SPECT-MR images 24 h post injection. SPECT images obtained 1 h post injection as well as 24 h post injection were fused with MR images, which showed that MBs and SPION can be utilized for multimodality imaging.

To establish the distribution of ^99m^Tc-labeled MBs, a reference containing free ^99m^Tc-pertechnetate was injected into the rat, and the distribution of radioactivity was scanned using SPECT/CT. The distribution of ^99m^Tc differed from that obtained after injection of the ^99m^Tc-labeled MBs. In rats injected with free ^99m^Tc-pertechnetate, the distribution was mainly to the stomach, kidneys, and bladder (Table 1). Some radioactivity was also observed in the thymus, thyroid gland, and mouth mucosal membranes. The different distribution observed after free ^99m^Tc-pertechnetate and ^99m^Tc-labeled MBs indicates that no free ^99m^Tc was present when injecting ^99m^Tc-labeled MBs. Radioactivity in the respective organs, measured by drawing ROIs on dynamic images, was in agreement with the visual evaluation of SPECT/CT images.

The high distribution of MBs into the lungs might be due to their size, lipophilicity, or building of aggregates with a diameter larger than 5 μm, causing them to be trapped in the capillary bed in the lungs. However, after 24 h, an accumulation of radioactivity was observed in the liver, which is most probably due to an uptake of MBs by the Kupffer cells. The fragments of the MBs might then be metabolized and eliminated through the kidneys and bladder as observed by SPECT/CT. It is also possible that this could be caused by the release of ^99m^Tc-pertechnetate *in vivo* from the ligands and/or through leakage from the PVA matrix over time.

^99m^Tc-tin colloids have been used as a radiopharmaceutical to measure the liver function. Kyung et al. injected both pigs and rats with ^99m^Tc-tin colloids to follow its biodistribution. The authors concluded from their investigation that in small animals (rats), the ^99m^Tc-tin colloids distributed exclusively to the liver directly post injection, while the distribution of colloids in the large animals (pigs) was observed in the lungs [31,32]. This, together with our results, indicates that our labeled MBs did not contain colloids due to the observed distribution to the lungs within the first hour. Moreover, most of the colloids could have been removed from the MBs through the several washing and centrifugation steps.

The use of SPION has been reported in several studies. Madru et al. reported that ^99m^Tc-labeled SPION was used to visualize lymph nodes in rats using SPECT/CT and MRI [7]. The authors also showed that SPION may be used both for MR as well as SPECT imaging when labeled by ^99m^Tc. In our study, the NOTA MBs containing SPION that were co-registered using SPECT/CT and MRI were mainly distributed to the lungs immediately post injection and redistributed to the liver and kidneys after 24 h. Lazarova et al. [4] labeled lipid shell ultrasound microbubbles from Visualsonics with ^99m^Tc and injected them into Wistar rats. The authors reported that the lipid MBs were accumulated in the liver and spleen after 4 and 60 min post administration. Using ultrasound contrast, it has also been demonstrated that not only the size, but also the surface architecture of the bubble influences the circulating time [33]. In the current study, the distribution was initially observed in the lungs after 60 min, but over time, radioactivity was gradually redistributed to the liver and spleen as well as to the kidneys and bladder. The different distribution pattern might be explained both by different MB composition and the attached ligands. One of the important factors for distribution is the amine content. It was recently demonstrated that the lung uptake was considerably increased by amine modifications [34]. Another factor that may influence adhesion properties, uptake, and impact on cells of MBs is the generation of a protein corona around nanoparticles [35] and MBs [36].

## Conclusions

In conclusion, fusion SPECT/CT imaging of ^99m^Tc-labeled MBs with various ligands is an appropriate way to study biodistribution and elimination *in vivo*. Moreover, incorporating SPION between the layers in MBs enables co-registration and multimodal imaging using SPECT/MRI and SPECT/CT. Each imaging technique gives an extra dimension to the information on biodistribution, elimination, and physiological status that enhances the interpretation of data. This in turn may increase the usefulness of multimodality in preclinical and clinical practice. A high labeling yield of DTPA-SPION MBs as well as their suitability for multimodal imaging SPECT/CT/MRI favor these MBs for further studies as a candidate for drug delivery of cytostatics and imaging of cancers such as lung cancer, HCC, or metastasis. Sufficient labeling yield of NOTA MBs, in combination with distribution to several organs including the lungs, liver and kidneys, favors this ligand as a candidate for future studies using PET. Further, the low lung-to-liver ratio after 24 h when using NOTA-SPION MBs indicates that the stiffening of MBs that is probably caused by the loading of SPION does not obstruct the redistribution from the pulmonary circulation to the liver.

## Competing interests

The authors declare that they have no competing interests.

## Authors’ contributions

ÅAB participated in the study design, carried out animal experiments, carried out ^99m^Tc labeling of microbubbles, performed MRI, performed SPECT/CT imaging, image reconstruction, data analysis, and drafted the manuscript. TBB participated in the study design and data analysis and drafted the manuscript. GE and LD participated in the study design, functionalized the microbubbles, and revised the manuscript. PA and GP participated in the study design and drafted the manuscript. LO and SM functionalized the microbubbles and revised the manuscript. AO performed SPECT/CT imaging and revised the manuscript. KC revised the manuscript. RA participated in the study design, performed visual evaluation, and revised the manuscript. MH designed the study, carried out animal experiments, performed data analysis, and finalized the manuscript. All authors read and approved the final manuscript.
